# Presence of neutrophil extracellular traps in bronchial aspirate of patients diagnosed with acute respiratory distress syndrome or acute exacerbation of idiopathic pulmonary fibrosis

**DOI:** 10.1186/cc14313

**Published:** 2015-03-16

**Authors:** M Ojima, N Yamamoto, T Hirose, S Hamaguchi, N Matsumoto, R Takegawa, M Seki, O Tasaki, K Tomono, T Shimazu

**Affiliations:** 1 Osaka University Graduate School of Medicine, Suita, Japan; 2 Nagasaki University Graduate School of Biomedical Sciences, Nagasaki, Japan

## Introduction

Neutrophil extracellular traps (NETs) are fibrous structures that are produced extracellularly from activated neutrophil. They can trap and kill various pathogens, and their release is one of the first lines of immune system. Meanwhile, recently it was reported that NETs also exert adverse effects in inflammatory diseases. Acute respiratory distress syndrome (ARDS) and acute exacerbation of idiopathic pulmonary fibrosis is acute-onset respiratory failure. It is characterized by excessive neutrophil infiltration into the alveolus, and great amounts of neutrophil elastase are released. The purpose of this study is to evaluate whether NETs are produced in bronchial aspirate of patients with ARDS or acute exacerbation of idiopathic pulmonary fibrosis, and to identify correlations with the respiratory function.

## Methods

This was a prospective observational study of seven patients admitted to the ICU of a large urban tertiary referral hospital. All patients were mechanically ventilated at the time of admission. The bronchial aspirates were collected serially from the patients by suction through a tracheal tube. To identify NETs, extracellular components including DNA, histone H3, and citrullinated histone H3 were simultaneously detected using immunohistochemistry. The respiratory function was evaluated by PaO_2_/FiO_2_ ratio (P/F ratio).

## Results

The study group was comprised of four men and three women. Median age was 69 (IQR: 61.0 to 72.5) years old. The reason for admission was ARDS (*n *= 5) and acute exacerbation of idiopathic pulmonary fibrosis (*n *= 2). The reasons for ARDS are bacterial pneumonia (*n *= 3), necrotizing fasciitis (*n *= 1) and extensive burn (*n *= 1). We identified NETs in the bronchial aspirates of all the patients. NET formation had persisted in four cases during the study period, their P/F ratio did not improve and all patients were dead due to respiratory failure. On the other hand, NETs decreased and vanished in three cases, their P/F ratio improved and all patients recovered from ARDS.

## Conclusion

NET formation was observed in bronchial aspirates of all the patients diagnosed with ARDS or acute exacerbation of idiopathic pulmonary fibrosis. It may be one of the prognostic factors of ARDS or acute exacerbation of idiopathic pulmonary fibrosis.

